# Supplementary material for: Development and validation of a functional assessment tool for Chinese inpatient rehabilitation: insights from a Delphi study based on the International Classification of Functioning, Disability, and Health (ICF)
Source: PeerJ. 2025 Nov 10;13:e20280. doi: 10.7717/peerj.20280 (PMC12614094; doi:10.7717/peerj.20280)
Supplement: Supplemental Information 2 [file peerj-13-20280-s002.docx]

Table S1. Results of the first round of the Delphi survey

| **ICF item** | **Numbers of individuals for each importance score** | | | | | **Mean** | **CV** |
| --- | --- | --- | --- | --- | --- | --- | --- |
|  | **5** | **4** | **3** | **2** | **1** |  |  |
| b130 Energy and drive functions | 10 | 4 | 0 | 0 | 1 | 4.467 | 0.237 |
| b134 Sleep functions | 8 | 5 | 1 | 1 | 0 | 4.333 | 0.208 |
| b152 Emotional functions | 4 | 6 | 5 | 0 | 0 | 3.933 | 0.203 |
| b280 Sensation of pain | 12 | 2 | 1 | 0 | 0 | 4.733 | 0.125 |
| b455 Exercise tolerance functions | 11 | 3 | 0 | 1 | 0 | 4.600 | 0.180 |
| b620 Urination functions | 10 | 4 | 0 | 0 | 1 | 4.467 | 0.237 |
| b640 Sexual functions | 0 | 0 | 6 | 1 | 8 | 1.867 | 0.531 |
| b710 Mobility of joint functions | 3 | 1 | 7 | 2 | 2 | 3.067 | 0.417 |
| b730 Muscle power functions | 5 | 8 | 0 | 2 | 0 | 4.067 | 0.236 |
| d230 Carrying out daily routine | 7 | 3 | 3 | 2 | 0 | 4.000 | 0.283 |
| d240 Handling stress and other psychological demands | 1 | 3 | 5 | 2 | 4 | 2.667 | 0.484 |
| d410 Changing basic body position | 10 | 3 | 0 | 2 | 0 | 4.400 | 0.240 |
| d415 Maintaining a body position | 2 | 1 | 2 | 4 | 6 | 2.267 | 0.634 |
| d420 Transferring oneself | 1 | 4 | 3 | 1 | 6 | 2.533 | 0.575 |
| d450 Walking | 14 | 1 | 0 | 0 | 0 | 4.933 | 0.052 |
| d455 Moving around | 3 | 1 | 2 | 3 | 6 | 2.467 | 0.648 |
| d465 Moving around using equipment | 5 | 3 | 1 | 3 | 3 | 3.267 | 0.497 |
| d470 Using transportation | 0 | 1 | 1 | 3 | 10 | 1.533 | 0.597 |
| d510 Washing oneself | 10 | 2 | 2 | 0 | 1 | 4.333 | 0.271 |
| d520 Caring for body parts | 6 | 2 | 1 | 5 | 1 | 3.467 | 0.434 |
| d530 Toileting | 11 | 1 | 1 | 0 | 2 | 4.267 | 0.337 |
| d540 Dressing | 11 | 2 | 1 | 0 | 1 | 4.467 | 0.252 |
| d550 Eating | 10 | 4 | 0 | 0 | 1 | 4.467 | 0.237 |
| d570 Looking after one's health | 1 | 5 | 5 | 1 | 3 | 3.000 | 0.418 |
| d640 Doing housework | 6 | 0 | 5 | 1 | 3 | 3.333 | 0.477 |
| d660 Assisting others | 3 | 2 | 2 | 5 | 3 | 2.800 | 0.526 |
| d710 Basic interpersonal interactions | 5 | 4 | 4 | 2 | 0 | 3.800 | 0.285 |
| d770 Intimate relationships | 2 | 1 | 2 | 6 | 4 | 2.400 | 0.563 |
| d850 Remunerative employment | 0 | 3 | 0 | 2 | 10 | 1.733 | 0.705 |
| d920 Recreation and leisure | 3 | 4 | 1 | 2 | 5 | 2.867 | 0.573 |
| Additional new items | No | | | | | | |

Note: gray markers indicate an mean important score ＜3.5

Table S2. Results of the second round of the Delphi survey

| **ICF item** | **Numbers of individuals for each importance score** | | | | | **Mean** | **CV** |
| --- | --- | --- | --- | --- | --- | --- | --- |
|  | **5** | **4** | **3** | **2** | **1** |  |  |
| b130 Energy and drive functions | 13 | 2 | 0 | 0 | 0 | 4.867 | 0.072 |
| b134 Sleep functions | 12 | 2 | 1 | 0 | 0 | 4.733 | 0.125 |
| b152 Emotional functions | 8 | 7 | 0 | 0 | 0 | 4.533 | 0.114 |
| b280 Sensation of pain | 12 | 2 | 1 | 0 | 0 | 4.733 | 0.125 |
| b455 Exercise tolerance functions | 9 | 5 | 1 | 0 | 0 | 4.533 | 0.141 |
| b620 Urination functions | 13 | 1 | 1 | 0 | 0 | 4.800 | 0.117 |
| b710 Mobility of joint functions | 2 | 5 | 4 | 2 | 2 | 3.200 | 0.395 |
| b730 Muscle power functions | 6 | 8 | 1 | 0 | 0 | 4.333 | 0.142 |
| d230 Carrying out daily routine | 8 | 4 | 3 | 0 | 0 | 4.333 | 0.188 |
| d410 Changing basic body position | 10 | 4 | 1 | 0 | 0 | 4.600 | 0.137 |
| d450 Walking | 14 | 1 | 0 | 0 | 0 | 4.933 | 0.052 |
| d465 Moving around using equipment | 3 | 6 | 0 | 4 | 2 | 3.267 | 0.440 |
| d510 Washing oneself | 12 | 3 | 0 | 0 | 0 | 4.800 | 0.086 |
| d520 Caring for body parts | 3 | 6 | 0 | 3 | 3 | 3.143 | 0.497 |
| d530 Toileting | 10 | 5 | 0 | 0 | 0 | 4.667 | 0.105 |
| d540 Dressing | 12 | 2 | 0 | 0 | 1 | 4.600 | 0.229 |
| d550 Eating | 13 | 1 | 1 | 0 | 0 | 4.800 | 0.117 |
| d570 Looking after one's health | 2 | 4 | 2 | 5 | 2 | 2.933 | 0.455 |
| d640 Doing housework | 0 | 0 | 3 | 6 | 6 | 1.800 | 0.430 |
| d710 Basic interpersonal interactions | 5 | 8 | 0 | 2 | 0 | 4.067 | 0.236 |

Note: gray markers indicate an mean important score ＜3.5

Table S3. Results of the third round of the Delphi survey

| **ICF item** | **Numbers of individuals for each importance score** | | | | | **Mean** | **CV** |
| --- | --- | --- | --- | --- | --- | --- | --- |
|  | **5** | **4** | **3** | **2** | **1** |  |  |
| b130 Energy and drive functions | 12 | 2 | 0 | 0 | 0 | 4.857 | 0.075 |
| b134 Sleep functions | 12 | 1 | 1 | 0 | 0 | 4.786 | 0.121 |
| b152 Emotional functions | 8 | 6 | 0 | 0 | 0 | 4.571 | 0.112 |
| b280 Sensation of pain | 12 | 2 | 0 | 0 | 0 | 4.857 | 0.075 |
| b455 Exercise tolerance functions | 9 | 4 | 1 | 0 | 0 | 4.571 | 0.141 |
| b620 Urination functions | 13 | 1 | 0 | 0 | 0 | 4.929 | 0.054 |
| b710 Mobility of joint functions | 4 | 4 | 3 | 1 | 2 | 3.500 | 0.400 |
| b730 Muscle power functions | 6 | 7 | 1 | 0 | 0 | 4.357 | 0.145 |
| d230 Carrying out daily routine | 8 | 3 | 3 | 0 | 0 | 4.357 | 0.193 |
| d410 Changing basic body position | 9 | 4 | 1 | 0 | 0 | 4.571 | 0.141 |
| d450 Walking | 13 | 1 | 0 | 0 | 0 | 4.929 | 0.054 |
| d465 Moving around using equipment | 5 | 5 | 0 | 2 | 2 | 3.643 | 0.412 |
| d510 Washing oneself | 11 | 3 | 0 | 0 | 0 | 4.786 | 0.089 |
| d520 Caring for body parts | 5 | 3 | 1 | 2 | 3 | 3.357 | 0.490 |
| d530 Toileting | 9 | 5 | 0 | 0 | 0 | 4.643 | 0.107 |
| d540 Dressing | 11 | 2 | 0 | 0 | 1 | 4.571 | 0.238 |
| d550 Eating | 13 | 1 | 0 | 0 | 0 | 4.929 | 0.054 |
| d710 Basic interpersonal interactions | 5 | 8 | 1 | 0 | 0 | 4.286 | 0.143 |

Note: gray markers indicate an mean important score ＜3.5
