# Supplementary material for: Development and validation of a functional assessment tool for Chinese inpatient rehabilitation: insights from a Delphi study based on the International Classification of Functioning, Disability, and Health (ICF)
Source: PeerJ. 2025 Nov 10;13:e20280. doi: 10.7717/peerj.20280 (PMC12614094; doi:10.7717/peerj.20280)
Supplement: Supplemental Information 3 [file peerj-13-20280-s003.docx]

# ICF Clinical Functioning Assessment Manual

The International Classification of Functioning, Disability and Health (ICF) is the third clinical outcome indicator proposed by the World Health Organization (WHO) in 2016, focusing specifically on functioning. Improvements or deteriorations in functioning can be quantitatively measured, thereby continuously indicating the state of medical efficacy and dynamically reflecting the quality of medical care. In 2001, WHO officially introduced the ICF at the World Health Assembly. As the second medical dictionary subsequent to the International Classification of Diseases (ICD), the ICF has seen widespread international application, particularly in areas such as clinical assessment, quality control, health insurance reimbursement standards, and economic benefit analysis, showcasing significant potential for further use. The Nanjing Health Insurance Rehabilitation Disease Group’s payment model research team convened rehabilitation experts in Nanjing, and conducted multiple discussions and clinical explorations. Based on the ICF Rehabilitation Set, they established a tool for clinical functioning assessment in rehabilitation departments, selecting 17 items through expert consensus and naming it ICF-RS-17. This functioning assessment manual is intended for use in clinical function assessments within rehabilitation departments.

## Clinical functioning assessment for ICF-RS-17

### Assessment content

| Body functions | |
| --- | --- |
| b130 Energy and drive functions | |
| Item description: | General mental functions of physiological and psychological mechanisms that cause the individual to move towards satisfy specific needs and general goals in a persistent manner. |
| Inclusions: | functions of energy level, motivation, appetites, craving (including craving for substances that can be abused), and impulse control |
| Exclusions: | consciousness functions; sleep functions; psychomotor functions; emotional functions |
| b134 Sleep functions | |
| Item description: | Can selectively sleep and maintain appropriate time and quality to meet daily needs. |
| Inclusions: | functions of amount of sleeping, and onset, maintenance and quality of sleep; functions involving the sleep cycle, such as in insomnia, hypersomnia and narcolepsy |
| Exclusions: | consciousness functions; energy and drive functions; attention function; psychomotor functions |
| b152 Emotional functions | |
| Item description: | The ability of an individual to generate appropriate emotions and manage various different emotions. |
| Inclusions: | functions of appropriateness of emotion, regulation and range of emotion; affect, sadness, happiness, love, fear, anger, hate, tension, anxiety, joy, sorrow; lability of emotion; flattening of affect |
| Exclusions: | temperament and personality functions; energy and drive functions |
| b280 Sensation of pain | |
| Item description: | An unpleasant subjective feeling related to emotion, specifically referring to persistent pain in a quiet state. |
| Inclusions: | sensations of generalized or localized pain, in one or more body part, pain in a dermatome, stabbing pain, burning pain, dull pain, aching pain; impairments such as myalgia, analgesia and hyperalgesia |
| b455 Exercise tolerance functions | |
| Item description: | The ability to sustain exercise for a certain duration and intensity. |
| Inclusions: | functions of physical endurance, aerobic capacity, stamina and fatiguability |
| Exclusions: | functions of the cardiovascular system; haematological system functions; respiration functions; respiratory muscle functions; additional respiratory functions |
| b620 Urination functions | |
| Item description: | The ability to voluntarily control and expel urine. |
| Inclusions: | functions of urination, frequency of urination, urinary continence; impairments such as in stress, urge, reflex, overflow, continuous incontinence, dribbling, automatic bladder, polyuria, urinary retention and urinary urgency |
| Exclusions: | urinary excretory functions; sensations associated with urinary functions |
| b710 Mobility of joint functions | |
| Item description: | Functions of the range and ease of movement of a joint. |
| Inclusions: | functions of mobility of single or several joints, vertebral, shoulder, elbow, wrist, hip, knee, ankle, small joints of hands and feet; mobility of joints generalized; impairments such as in hypermobility of joints, frozen joints, frozen shoulder, arthritis |
| Exclusions: | stability of joint functions; control of voluntary movement functions |
| b730 Muscle power functions | |
| Item description: | Functions related to the force generated by the contraction of a muscle or muscle groups. |
| Inclusions: | functions associated with the power of specific muscles and muscle groups, muscles of one limb, one side of the body, the lower half of the body, all limbs, the trunk and the body as a whole; impairments such as weakness of small muscles in feet and hands, muscle paresis, muscle paralysis, monoplegia, hemiplegia, paraplegia, quadriplegia and akinetic mutism |
| Exclusions: | muscle tone functions; muscle endurance functions |
| General tasks and demands | |
| d230 Carrying out daily routine | |
| Item description: | Planning, organizing, and completing daily tasks. |
| Inclusions: | Three types of tasks: 1. Upper limb-related tasks, such as doing laundry; 2. Calculation-related tasks, such as shopping at the supermarket and making change; 3. Planned tasks, such as brushing teeth, which involves squeezing toothpaste, filling the toothbrush with water, and rinsing. |
| Mobility | |
| d410 Changing basic body position | |
| Item description: | Transitioning from one body position to another. |
| Inclusions: | changing body position from lying down, from squatting or kneeling, from sitting or standing, rolling over, bending and shifting the body's centre of gravity |
| Exclusions: | transferring oneself |
| d450 Walking | |
| Item description: | Moving along a surface on foot, step by step, so that one foot is always on the ground |
| Inclusions: | walking with assistance from others and using assistive devices, whether for short distances or long distances |
| Exclusions: | transferring oneself; moving around |
| d465 Moving around using equipment | |
| Item description: | Using specially designed equipment (such as a wheelchair) to move the body from one place to another |
| Exclusions: | transferring oneself; walking; using transportation; driving |
| Self-care | |
| d510 Washing oneself | |
| Item description: | Washing and drying the body, such as taking a shower, washing hands, washing feet, washing face, and washing hair. |
| Inclusions: | washing body parts, the whole body, and drying oneself |
| Exclusions: | caring for body parts; toileting |
| d530 Toileting | |
| Item description: | Planning and carrying out the elimination of human waste (menstruation, urination and defecation), and clean oneself afterwards. |
| Inclusions: | regulating urination, defecation and menstrual care |
| Exclusions: | washing oneself; caring for body parts |
| d540 Dressing | |
| Item description: | Choosing clothing and footwear based on the climate and environment, and putting them on and taking them off in an appropriate manner. |
| Inclusions: | putting on or taking off clothes and footwear and choosing appropriate clothing |
| d550 Eating | |
| Item description: | Using appropriate utensils to bring food to the mouth and being able to swallow it. |
| Exclusions: | drinking |
| Interpersonal interactions and relationships | |
| d710 Basic interpersonal interactions | |
| Item description: | Interacting with people in a contextually and socially appropriate manner. |
| Inclusions: | showing respect, warmth, appreciation, and tolerance in relationships; responding to criticism and social cues in relationships; and using appropriate physical contact in relationships |

### **Assessment method**

#### Numerical Rating Scale

The quantification of functional level uses the Numerical Rating Scale (NRS). The NRS has two ends, which are 0 and 10, where 0 represents completely normal and 10 represents complete impairment (Figure 3).

completely normal complete impairment

**0 1 2 3 4 5 6 7 8 9 10**

Figure 3. Numerical Rating Scale

#### The application of NRS in disability level assessment

This standard divides the NRS from 0 to 10 into five levels: completely normal (0 points), mild impairment (1-3 points), moderate impairment (4-6 points), severe impairment (7-9 points), and complete obstruction (10 points). Items related to mobility/activity/participation are evaluated by the assessor based on the performance of the subject in completing tasks. In contrast, evaluations related to sleep, emotions, pain, etc., are based on the self-perception of the assessed individual.

#### General Principles of Assessment

Although this user manual makes a broad classification of disability levels in the NRS assessment into five categories: normal, mild impairment, moderate impairment, severe impairment, and complete obstruction, it is important to note that, apart from the normal and complete obstruction levels which do not require further subdivision, the other three levels—mild, moderate, and severe impairment—still need further subdivision within the same broad category during actual assessments. For instance, mild impairment must be defined as 1-3 points. The degree of a patient’s functional impairment can be defined by the amount of assistance provided by others, the patient’s efficiency and extent in completing a task, and the patient’s subjective feelings of fatigue. To better guide practice, an explanation of the general principles of assessment is provided before the NRS rating begins, to help users better understand the scoring criteria for each item.

- For those who are unable to be assessed due to consciousness disorders, hearing comprehension difficulties, or insufficient clinical data that may cause harm to the evaluator and assessment procedures (such as strength assessment in fracture patients), mark as NA and state the reason.
- If there is still potential for improvement in the functional assessment of each item, the score for that item shall not be 0 points, in order to reflect functional improvements after intervention.
- In the classification of mild, moderate, and severe impairment, ‘mild impairment’ requires supervision or guidance from others (non-contact assistance) or the ability to complete tasks independently using assistive devices; ‘moderate impairment’ requires minimal contact assistance or help to complete less than half of the activity; ‘severe impairment’ requires substantial contact assistance or help to complete half or more of the activity.
- In the classification of mild, moderate, and severe impairment, ‘mild impairment’ requires supervision or guidance from others (non-contact assistance) or the ability to complete tasks independently using assistive devices; ‘moderate impairment’ requires minimal contact assistance or help to complete less than half of the activity; ‘severe impairment’ requires substantial contact assistance or help to complete half or more of the activity.
- Minimal contact assistance is defined as help provided by one person that requires little effort or assistance that can be performed with one hand; substantial contact assistance involves help that is labor-intensive by one or both hands or requires two people using one hand.
- The degree of functional impairment can be evaluated based on efficiency, safety, and completion extent while performing tasks. For example, “d450 Walking” may indicate mild impairment, which can be further refined to 1-3 points based on distance, speed, and safety of walking.
- For the assessment of the degree of impairment in certain items, patients can define their level of completion for the task related to the item, such as d510 Washing oneself, d530 Toileting, and d540 Dressing.
- Functional impairment assessment can also reference the subjective degree of effort required to complete tasks.
- During the assessment process, choose either “d450 Walking” or “d465 Moving around” using equipment for assessment; patients capable of walking should not be assessed for moving around with tools.

#### NRS assessment based on task execution performance

When assessing the items related to activity ability, personal care, and social/interpersonal communication in the assessment of disability levels, the assessment is primarily based on the individual’s performance when executing the above items. The items related to these areas are shown in Table 1.

Table 1. Items for NRS Assessment Based on Task Execution Performance

| **Domain** | **Item** |
| --- | --- |
| Activity ability | d450 Walking  b455 Exercise tolerance functions  d465 Moving around using equipment  d410 Changing basic body position |
| Personal care | b620 Urination functions  d230 Carrying out daily routine  d510 Washing oneself  d530 Toileting  d540 Dressing  d550 Eating |
| Muscle strength and joint mobility | b710 Mobility of joint functions  b730 Muscle power functions |
| Social/interpersonal communication | d710 Basic interpersonal interactions |
| Mental and psychological functions | b130 Energy and drive functions |

#### NRS assessment based on individual subjective feelings

For the items related to sleep function, emotional function, pain perception, and other areas in the assessment of disability levels, the actual assessment is primarily based on the individual’s subjective experience in these areas. The items related to these areas are shown in Table 2. To evaluate these items, the individual’s subjective feelings regarding these functional areas need to be inquired about, and the individual rates their experience using the NRS based on their own subjective feelings. For example, in the pain numerical rating scale, the assessor explains the meanings of the items and the significance of scores 0 and 10, allowing the individual to easily judge their own pain level. The NRS can be used in the same manner for assessing b134 sleep function and b152 emotional function items. During the assessment, the assessor should adequately explain the meanings of the items to the patient, while also considering the assessment of the patient’s functional state to make a final assessment. If the individual is unable to communicate normally with the assessor due to reasons such as consciousness impairment, cognitive impairment, or speech impairment, these items can be marked as unable to assess.

Table 2. Items for NRS Assessment Based on Individual Subjective Experience

| **Domain** | **Item** | |
| --- | --- | --- |
| Sleep | ⚫ | b134 Sleep functions |
| Emotion | ⚫ | b152 Emotional functions |
| Pain perception | ⚫ | b280 Sensation of pain |

#### The detailed classification of levels for ICF items

**d450 Walking**

Item description: Moving along a surface on foot, step by step, so that one foot is always on the ground

Inclusions: Walking with assistance from others and using assistive devices, whether for short distances or long distances

Exclusions: Transferring oneself; moving around

Assessment Prompt: How has your ability to walk been in the past two days? How many meters can you walk on level ground?

Assessment Guidance: This pertains to assessing the evaluator’s ability to walk on level ground. The assessed individual may wear braces or prosthetics, or use walking aids.

Assessment Criteria:

**0 Points:** The individual can walk independently on level ground for more than 400 meters without the risk of falling.

**1-3 Points:** The individual can walk independently on level ground, but there is a risk of falling; they require supervision from others or the use of assistive devices such as a cane or walker.

**1 Point:** Requires supervision to walk and can walk 100-400 meters.

**2 Points:** Requires supervision to walk and can walk 51-100 meters.

**3 Points:** Requires supervision to walk and can walk 50 meters.

**4-6 Points:** The individual requires minimal assistance (one person) when walking 30 meters.

**4 Points:** Slight assistance with one hand (less than 30%).

**5 Points:** Assistance with one hand (31%-70%).

**6 Points:** Assistance with one hand (≥71%).

**7-9 Points:** The individual requires substantial assistance (two-handed or two-person) when walking 30 meters.

**7 Points:** Slight assistance with both hands (less than 30%).

**8 Points:** Assistance with both hands (31%-70%).

**9 Points:** Assistance with both hands (71%-99%).

**10 Points:** Unable to walk and completely dependent on others.

**d465 Moving around using equipment**

Item description: Using specially designed equipment (such as a wheelchair) to move the body from one place to another

Exclusions: transferring oneself; walking; using transportation; driving

Assessment Prompt: How has your ability to move from one place to another using equipment (such as a wheelchair, electric scooter, etc.) been in the past two days?

Assessment Guidance: This assessment item evaluates the individual’s ability to manipulate mobility devices (such as a wheelchair) to assist themselves in moving from one location to another. Note that walking functionality is not included.

Remark: If the patient has the ability to walk, this item will not be assessed.

Assessment Criteria:

**0 Points:** The individual can independently use a wheelchair to move from one place to another for at least 400 meters.

**1-3 Points:** The individual requires supervision or guidance from others while using a wheelchair to move.

**1 Point:** Outdoor movement exceeds 100 meters.

**2 Points:** Outdoor movement is 50-100 meters.

**3 Points:** Indoor movement.

**4-6 Points:** The individual requires minimal contact assistance from others (or needs help less than 50% of the time) while using a wheelchair to move.

**4 Points:** Less than 10% of the process of driving the wheelchair requires help from others.

**5 Points:** 11-30% of the process of driving the wheelchair requires help from others.

**6 Points:** 31-50% of the process of driving the wheelchair requires help from others.

**7-9 Points:** The individual requires substantial contact assistance from others (or needs help more than 50% of the time) while using a wheelchair to move.

**7 Points:** 51-60% of the process of driving the wheelchair requires help from others.

**8 Points:** 61-80% of the process of driving the wheelchair requires help from others.

**9 Points:** 81-99% of the process of driving the wheelchair requires help from others.

**10 Points:** The individual completes mobility with equipment entirely dependent on others.

**b455 Exercise tolerance functions**

Item description: The ability to sustain exercise for a certain duration and intensity.

Inclusions: functions of physical endurance, aerobic capacity, stamina and fatiguability

Exclusions: functions of the cardiovascular system; haematological system functions; respiration functions; respiratory muscle functions; additional respiratory functions

Assessment Prompt: How long were you able to engage in active training each day in the past two days? Please evaluate based on your current active training situation.

Assessment Criteria:

**0 Points:** Normal active training with no fatigue.

**1-3 Points:** Able to complete 3 hours (180 minutes) or more of active training, with occasional feelings of fatigue.

**1 Point:** Completed more than 180 minutes of active training and can recover with 5-10 minutes of rest.

**2 Points:** Completed more than 180 minutes of active training and can recover with 11-20 minutes of rest.

**3 Points:** Completed more than 180 minutes of active training and requires more than 20 minutes of rest to recover.

**4-6 Points:** Able to complete 1-3 hours of active training, with frequent fatigue.

**4 Points:** Able to complete 121-180 minutes of active training.

**5 Points:** Able to complete 91-120 minutes of active training.

**6 Points:** Able to complete 60-90 minutes of active training.

**7-9 Points:** Able to complete less than 1 hour of active training, often feeling very fatigued and unable to complete some daily activities continuously.

**7 Points:** Able to complete only 40-59 minutes of active training.

**8 Points:** Able to complete only 20-39 minutes of active training.

**9 Points:** Able to complete less than 20 minutes of active training.

**10 Points:** Unable to complete active training, very easily fatigued.

**b620 Urination functions**

Item description: The ability to voluntarily control and expel urine.

Inclusions: functions of urination, frequency of urination, urinary continence; impairments such as in stress, urge, reflex, overflow, continuous incontinence, dribbling, automatic bladder, polyuria, urinary retention and urinary urgency

Exclusions: urinary excretory functions; sensations associated with urinary functions

Assessment Prompt: In the past two days, have you experienced any issues with urination? Have you had an increased frequency of urination, urinary incontinence, or difficulty urinating?

Assessment Guidance: Urination issues include increased frequency of urination, urinary retention, and urinary incontinence.

Assessment Criteria:

**0 Points:** The individual can control urination independently, with normal frequency and control.

**1-3 Points:** The individual can control urination during the day, but experiences increased frequency at night, or uses assistive devices such as diapers or pads; or can perform intermittent clean intermittent catheterization independently or under supervision or prompting.

**1 Point:** With normal fluid intake at night, the individual has an increased urination frequency of 2-3 times over several days; can independently complete intermittent catheterization.

**2 Points:** Experiences urination 4-5 times at night; may need intermittent supervision/comments to complete intermittent catheterization independently.

**3 Points:** Experiences urination more than 5 times at night; or completes intermittent catheterization independently with full supervision.

**4-6 Points:** The individual can generally control urination independently during the day, with occasional (less than once per day but more than once per week) instances of urinary incontinence; has poorer control at night, or requires minimal assistance from others to use devices such as diapers or pads; or can perform intermittent clean intermittent catheterization with minimal assistance from others.

**4 Points:** Experiences urinary incontinence 1-2 times a week; or receives less than 10% assistance from others during the urination or intermittent catheterization process.

**5 Points:** Experiences urinary incontinence 3-4 times a week; or receives 11-30% assistance from others during the urination or intermittent catheterization process.

**6 Points:** Experiences urinary incontinence 5-6 times a week; or receives 31-50% assistance from others during the urination or intermittent catheterization process.

**7-9 Points:** The individual is mostly unable to control urination during the day (1 or more times per day, but not completely out of control), experiences urinary incontinence at night, or requires substantial assistance from others to use devices such as diapers or pads; or can perform intermittent clean intermittent catheterization with substantial assistance from others.

**7 Points:** Experiences urinary incontinence once per day; or receives 51-60% assistance from others during the urination or intermittent catheterization process.

**8 Points:** Experiences urinary incontinence twice per day; or receives 61-80% assistance from others during the urination or intermittent catheterization process.

**9 Points:** Experiences urinary incontinence three times per day; or receives 81-99% assistance from others during the urination or intermittent catheterization process.

**10 Points:** Experiences urinary incontinence, is completely unable to control urination, has an indwelling catheter, or is entirely dependent on others.

**b710 Mobility of joint functions**

Item description: Functions of the range and ease of movement of a joint.

Inclusions: functions of mobility of single or several joints, vertebral, shoulder, elbow, wrist, hip, knee, ankle, small joints of hands and feet; mobility of joints generalized; impairments such as in hypermobility of joints, frozen joints, frozen shoulder, arthritis

Exclusions: stability of joint functions; control of voluntary movement functions

Assessment Prompt: Have your joint ranges of motion been limited in the past two days?

Assessment Guidance: This item evaluates the passive range of motion (PROM) of affected joints, primarily including passive movements of the shoulder, elbow, wrist, hip, knee, and ankle on the affected side. The assessment can focus on the joints with the most significant functional impairments based on the patient’s diagnosis and functional status.

Remark: It is recommended that this item be assessed by a rehabilitation therapist, focusing on the passive range of motion of the joints. This assessment primarily evaluates the range of motion in larger joints such as the shoulder, elbow, wrist, hip, knee, and ankle.

Assessment Criteria:

**0 Points:** No abnormalities in passive joint motion function.

**1-3 Points:** Mild impairment in the amplitude of passive joint motion (joint range of motion limited to ≤30%).

**1 Point:** The range of motion of the affected joint is limited to within 10%.

**2 Points:** The range of motion of the affected joint is limited to 11%-20%.

**3 Points:** The range of motion of the affected joint is limited to 21%-30%.

**4-6 Points:** Moderate impairment in the amplitude of passive joint motion (joint range of motion limited to 31%-60%).

**4 Points:** The range of motion of the affected joint is limited to 31%-40%.

**5 Points:** The range of motion of the affected joint is limited to 41%-50%.

**6 Points:** The range of motion of the affected joint is limited to 51%-60%.

**7-9 Points:** Severe impairment in the amplitude of passive joint motion (joint range of motion limited to 61%-90%).

**7 Points:** The range of motion of the affected joint is limited to 61%-70%.

**8 Points:** The range of motion of the affected joint is limited to 71%-80%.

**9 Points:** The range of motion of the affected joint is limited to 81%-90%.

**10 Points:** Extremely severe impairment in the amplitude of passive joint motion (joint range of motion limited to >90%).

Notes:

If the range of motion cannot be assessed due to early-stage fractures, mark it as NA.

If the patient has joint motion limitations in multiple areas, the detailed scoring can be determined based on the degree of limitation and the weight of the affected joints.

The directions of motion and normal ranges for each joint are referenced in the table below.

Affected joint weight = 1 / number of affected joints (upper limb includes the shoulder, elbow, and wrist joints; lower limb includes the hip, knee, and ankle joints).

Total score = Affected joint score * sum of affected joint weights.

The direction of movement and normal range of motion for joint range of motion measurement

| Shoulder Joint | Flexion | 0 °-180 ° |
| --- | --- | --- |
|  | Extension | 0 °-50 ° |
|  | Adduction | 0 °-45 ° |
|  | Abduction | 0 °-180 ° |
| Elbow Joint | Flexion | 0 °-150 ° |
|  | Extension | 0 ° |
| Wrist Joint | Palmar Flexion | 0 °-90 ° |
|  | Dorsal Extension | 0 °-70 ° |
| Hip Joint | Flexion | 0 °-125 ° |
|  | Extension | 0 °-15 ° |
|  | Abduction | 0 °-45 ° |
|  | Adduction | 0 °-45 ° |
| Knee Joint | Flexion | 0 °-150 ° |
|  | Extension | 0 ° |
| Ankle Joint | Dorsiflexion | 0 °-20 ° |
|  | Plantarflexion | 0 °-45 ° |
| Cervical Spine | Flexion | 0 °-45 ° |
|  | Extension | 0 °-45 ° |
|  | Lateral Flexion | 0 °-45 ° |
|  | Rotation | 0 °-80 ° |
| Thoracolumbar Spine | Flexion | 0 °-80 ° |
|  | Extension | 0 °-30 ° |
|  | Lateral Flexion | 0 °-40 ° |
|  | Rotation | 0 °-45 ° |

**b730 Muscle power functions**

Item description: Functions related to the force generated by the contraction of a muscle or muscle groups.

Inclusions: functions associated with the power of specific muscles and muscle groups, muscles of one limb, one side of the body, the lower half of the body, all limbs, the trunk and the body as a whole; impairments such as weakness of small muscles in feet and hands, muscle paresis, muscle paralysis, monoplegia, hemiplegia, paraplegia, quadriplegia and akinetic mutism

Exclusions: muscle tone functions; muscle endurance functions

Assessment Prompt: How is the functional strength of your muscles currently?

Assessment Guidance: The evaluator performs manual muscle assessment on the patient’s muscle strength, focusing only on the affected muscles (e.g., in patients with hemiplegia, the assessment will be on the upper and lower limbs on the affected side; in paraplegic patients, it will be on both lower limbs). The evaluator may select the primary muscle groups with functional impairments based on the patient’s diagnosis and functional status for assessment.

Note: This item is assessed by a rehabilitation therapist.

Assessment Scoring Criteria:

**0 Points:** Muscle strength is at Level 5 (MMT assessment), strength is normal.

**1-3 Points:** Muscle strength is at Level 4 (MMT assessment), able to resist partial resistance.

**1 Point:** 4+

**2 Points:** 4

**3 Points:** 4-

**4-6 Points:** Muscle strength is at Level 3 (MMT assessment), able to complete activities against gravity but cannot resist.

**4 Points:** 3+

**5 Points:** 3

**6 Points:** 3-

**7-9 Points:** Muscle strength is at Level 2 (MMT assessment), able to perform activities under gravity-eliminated conditions.

**7 Points:** 2+

**8 Points:** 2

**9 Points:** 2-

**10 Points:** Muscle strength is at Level 0 or 1 (MMT assessment), no active movement.

Note: If multiple areas are affected, scoring can be refined based on the location and degree of muscle involvement. For example, if a hemiplegic patient has muscle strength impairment in both the upper and lower limbs on the affected side, the overall score can be calculated with each limb weighting at 50%. For paraplegic patients, both sides of the limbs will also each count as 50% in the overall score.

Total Score Calculation Rules: The total score is calculated based on the muscle strength scores of the affected areas and their respective weights.

Affected area weight = 1 / Number of affected areas (the upper limb includes three areas: shoulder, elbow, wrist; the lower limb includes three areas: hip, knee, ankle)

Total Score = Muscle strength score for affected area * Weight of affected area

**d230 Carrying out daily routine**

Item description: Planning, organizing, and completing daily tasks.

Inclusions: Three types of tasks: 1. Upper limb-related tasks, such as doing laundry; 2. Calculation-related tasks, such as shopping at the supermarket and making change; 3. Planned tasks, such as brushing teeth, which involves squeezing toothpaste, filling the toothbrush with water, and rinsing.

Assessment Prompt: In the past two days, how would you assess your ability to independently handle daily activities (such as washing your face, making small purchases, brushing your teeth, etc.)?

Assessment Guidance: The assessment content of this item includes the assessed individual’s ability to plan, organize, and complete daily activities, including motor functions of the upper limbs and hands, as well as the ability to plan and organize procedural tasks. The steps for brushing teeth include: squeezing toothpaste (1), obtaining mouthwash (2), the process of brushing teeth (3), and cleaning the mouth (4); the steps for washing the face include: alternately rubbing the towel with both hands (1), washing the face in an appropriate manner (2), and wringing out the towel (3); small purchases include: online shopping (1), ordering takeout via a mobile phone (1), or elderly patients correctly calculating small amounts (1).

Assessment Criteria:

**0 Points:** The individual is fully independent in planning, organizing, and completing daily activities (the complete processes of washing the face, making small purchases, and brushing teeth) without assistance.

**1-3 Points:** The individual requires monitoring or guidance from others to plan, organize, and complete daily activities.

**1 Point:** 1%-30% of the process requires monitoring or reminders from others.

**2 Points:** 31%-60% of the process requires monitoring or reminders from others.

**3 Points:** 61%-100% of the process requires monitoring or reminders from others.

**4-6 Points:** The individual requires a small amount of help in planning, organizing, and completing daily activities (requiring less than half of the assistance during the process).

**4 Points:** Less than 10% of the process needs help from others, with one step needing assistance in washing the face, making small purchases, or brushing teeth.

**5 Points:** 11%-30% of the process needs help from others, with two steps needing assistance in washing the face, making small purchases, or brushing teeth.

**6 Points:** 31%-50% of the process needs help from others, with three steps needing assistance in washing the face, making small purchases, or brushing teeth.

**7-9 Points:** The individual needs a substantial amount of help in planning, organizing, and completing daily activities (requiring half or more of the assistance during the process).

**7 Points:** 51%-60% of the process needs help from others, with four to five steps needing assistance in washing the face, making small purchases, or brushing teeth.

**8 Points:** 61%-80% of the process needs help from others, with six to seven steps needing assistance in washing the face, making small purchases, or brushing teeth.

**9 Points:** 81%-99% of the process needs help from others, with only cooperative actions occurring, and eight steps needing assistance in washing the face, making small purchases, or brushing teeth.

**10 Points:** The individual is completely dependent on others for daily activities.

**d410 Changing basic body position**

Item description: Transitioning from one body position to another.

Inclusions: changing body position from lying down, from squatting or kneeling, from sitting or standing, rolling over, bending and shifting the body's centre of gravity

Exclusions: transferring oneself

Assessment Prompt: In the past two days, how would you assess your ability to change body positions?

Assessment Guidance: This assessment includes the ability to change positions from lying to sitting, from lying to standing, and from sitting to standing.

Assessment Criteria:

**0 Points:** Able to independently complete various position transfers without assistance from others.

**1-3 Points:** Requires supervision or assistance to complete transfers from sitting to standing.

**1 Point:** Can complete the sitting-to-standing transfer under supervision and guidance from others.

**2 Points:** Requires a small amount (<50%) of assistance for the sitting-to-standing transfer.

**3 Points:** Requires a large amount (≥50%) of assistance for the sitting-to-standing transfer.

**4-6 Points:** Requires supervision or assistance to complete transfers from lying to sitting (such as rolling to sit up or shifting the center of gravity while sitting), but cannot complete standing position transfers.

**4 Points:** Can complete the lying-to-sitting transfer under supervision or guidance from others.

**5 Points:** Requires a small amount (<50%) of assistance for the lying-to-sitting transfer.

**6 Points:** Requires a large amount (≥50%) of assistance for the lying-to-sitting transfer.

**7-9 Points:** Can complete rolling transfers from lying position with supervision or assistance but cannot complete sitting or standing position transfers.

**7 Points:** Can roll under supervision and guidance from others.

**8 Points:** Requires a small amount (<50%) of assistance to roll.

**9 Points:** Requires a large amount (≥50%) of assistance to roll.

**10 Points:** Unable to perform any actions to change body positions.

**d530 Toileting**

Item description: Planning and carrying out the elimination of human waste (menstruation, urination and defecation), and clean oneself afterwards.

Inclusions: regulating urination, defecation and menstrual care

Exclusions: washing oneself; caring for body parts

Assessment Prompt: In the past two days, have you been able to complete toilet use and post-use cleaning?

Assessment Guidance: The seven steps of using the toilet include sitting down on the toilet (1), standing up (2), pulling down (3) and putting on (4) pants, completing the elimination process (5), using toilet paper for personal cleaning (6), and flushing the toilet afterwards (7).

Assessment Criteria:

**0 Points:** The individual can independently complete the toilet use actions.

**1-3 Points:** The individual requires monitoring or guidance from others during toilet use but does not need physical assistance.

**1 Point:** 1%-30% of the process requires monitoring or reminders from others.

**2 Points:** 31%-60% of the process requires monitoring or reminders from others.

**3 Points:** 61%-100% of the process requires monitoring or reminders from others.

**4-6 Points:** The individual requires a small amount of physical assistance from others during toilet use (requiring help in less than half of the seven steps).

**4 Points:** 1 step of the toilet use process needs help from others.

**5 Points:** 2 steps of the toilet use process need help from others.

**6 Points:** 3 steps of the toilet use process need help from others.

**7-9 Points:** The individual requires a substantial amount of physical assistance from others during toilet use (requiring help in more than half of the steps).

**7 Points:** 4 steps of the toilet use process need help from others.

**8 Points:** 5 steps of the toilet use process need help from others.

**9 Points:** 6 steps of the toilet use process need help from others.

**10 Points:** The individual is completely dependent on others for toilet use.

**d510 Washing oneself**

Item description: Washing and drying the body, such as taking a shower, washing hands, washing feet, washing face, and washing hair.

Inclusions: washing body parts, the whole body, and drying oneself

Exclusions: caring for body parts; toileting

Assessment Prompt: In the past two days, have you been able to wash and dry various parts of your body?

Assessment Guidance: This assessment includes cleaning, rinsing, and drying the areas from the neck to the feet. The nine parts of the body you should wash and dry include the head (1), neck and face (2), both upper limbs (3), both hands (4), chest and abdomen (5), back (6), both lower limbs (7), both feet (8), and perineum (9).

Assessment Criteria:

**0 Points:** The individual can independently wash all parts of their body (the nine parts).

**1-3 Points:** The individual requires assistance with preparation or cleanup but can complete the washing and drying; or may need monitoring from others to ensure safety, without requiring physical assistance.

**1 Point:** 1%-30% of the process requires monitoring or reminders from others.

**2 Points:** 31%-60% of the process requires monitoring or reminders from others.

**3 Points:** 61%-100% of the process requires monitoring or reminders from others.

**4-6 Points:** The individual requires a small amount of physical assistance from others during washing (requiring help in less than half of the process).

**4 Points:** 1 part of the washing process requires help from others.

**5 Points:** 2 parts of the washing process require help from others.

**6 Points:** 3 parts of the washing process require help from others.

**7-9 Points:** The individual requires a substantial amount of physical assistance from others during washing (requiring help in more than half of the process).

**7 Points:** 4-5 parts of the washing process require help from others.

**8 Points:** 6-7 parts of the washing process require help from others.

**9 Points:** 8-9 parts of the washing process require help from others.

**10 Points:** The individual is completely dependent on others for washing their body.

**d540 Dressing**

Item description: Choosing clothing and footwear based on the climate and environment, and putting them on and taking them off in an appropriate manner.

Inclusions: putting on or taking off clothes and footwear and choosing appropriate clothing

Assessment Prompt: In the last two days, how well have you been able to put on and take off clothing?

Assessment Guidelines: The assessment for this item includes putting on, taking off, and fastening clothing; it also includes orthotics and prosthetics if needed. The six steps for dressing and undressing are: putting on a shirt (1), putting on pants (2), taking off a shirt (3), taking off pants (4), putting on and taking off socks (5), putting on and taking off shoes (6).

Assessment Details:

**0 Points:** The individual can independently and appropriately complete the process of putting on and taking off clothing and shoes/socks without needing help from others.

**1-3 Points:** The individual requires supervision or guidance from others when putting on and taking off clothing and shoes/socks, but does not require physical assistance.

**1 Point:** 1%-30% of the process requires supervision or reminders from others.

**2 Points:** 31%-60% of the process requires supervision or reminders from others.

**3 Points:** 61%-100% of the process requires supervision or reminders from others.

**4-6 Points:** The individual can dress and undress most clothing independently, but some clothing (either a shirt, pants, or shoes/socks) requires assistance from others, or they cannot complete certain fine motor tasks (such as buttoning, tying shoelaces, pulling up zippers, etc.) independently and require minimal physical assistance, meaning they still need help from others for less than half of the process.

**4 Points:** Can complete the six dressing steps, but cannot independently perform some fine motor tasks such as buttoning or pulling up zippers and needs help from others; or requires assistance for one step during the dressing process.

**5 Points:** Requires assistance for two steps during the dressing process.

**6 Points:** Requires assistance for three steps during the dressing process.

**7-9 Points:** The individual can only independently dress and undress a small portion of clothing and requires extensive physical assistance from others, meaning they need assistance for half or more of the activities.

**7 Points:** Requires assistance for four steps during the dressing process.

**8 Points:** Requires assistance for five steps during the dressing process.

**9 Points:** Requires assistance for all six steps during the dressing process, with the individual being only slightly cooperative.

**10 Points:** The individual is completely reliant on others for dressing and undressing clothing and shoes/socks and is unable to cooperate.

**d550 Eating**

Item description: Using appropriate utensils to bring food to the mouth and being able to swallow it.

Exclusions: drinking

Assessment Prompt: In the last two days, have you been able to eat independently?

Assessment Guidelines: This assessment includes the ability to use appropriate utensils to bring food to the mouth and swallow, including feeding, chewing, and swallowing functions. It does not include the meal preparation process.

Assessment Details:

**0 Points:** The individual can independently use utensils to bring food to their mouth and swallow without choking.

**1-3 Points:** The individual requires supervision or instructions during eating, or independently uses aids or intermittently relies on tube feeding.

**1 Point:** 1%-30% of the eating process requires supervision or reminders from others.

**2 Points:** 31%-60% of the eating process requires supervision or reminders from others.

**3 Points:** 61%-100% of the eating process requires supervision or reminders from others.

**4-6 Points:** The individual requires minimal physical assistance during eating. They still need help from others for less than half of the process, or occasional choking (less than once a day but more than once a week).

**4 Points:** Requires help from others during less than 10% of the eating process or intermittent tube feeding, or chokes twice a week.

**5 Points:** Requires help from others during 11-30% of the eating process or intermittent tube feeding, or chokes 3-4 times a week.

**6 Points:** Requires help from others during 31-50% of the eating process or intermittent tube feeding, or chokes 5-6 times a week.

**7-9 Points:** The individual requires significant physical assistance during the eating process; more than half of the activities require help from others or experiences frequent choking.

**7 Points:** Requires help from others during 51-60% of the eating process or tube feeding, or chokes once per meal.

**8 Points:** Requires help from others during 61-80% of the eating process or tube feeding, or chokes twice per meal.

**9 Points:** Requires help from others during 81%-99% of the eating process or tube feeding, or chokes three times per meal.

**10 Points:** The individual is completely reliant on others for eating assistance, has difficulty swallowing, or has a feeding tube in place.

**d710 Basic interpersonal interactions**

Item description: Interacting with people in a contextually and socially appropriate manner.

Inclusions: showing respect, warmth, appreciation, and tolerance in relationships; responding to criticism and social cues in relationships; and using appropriate physical contact in relationships

Assessment Prompt: In the last two days, how has your communication and interaction with others been?

Assessment Guidelines: Evaluators can communicate with the patient through the following questions and make judgments based on the nature of the communication with the patient (including aspects like initiative, appropriateness, language organization ability, and expression skills). The assessment primarily involves the following five dimensions: fluency of expression, listening comprehension ability, execution of instructions, repetition, and naming.

This item’s score is the total sum of the scores from the following five dimensions.

| **Domain** | **Question (Reference)** | **Score** | **Score Prompt** |
| --- | --- | --- | --- |
| **1.** **Fluency of Expression: Answer the following questions.** | What is your name? | 0 | The responses to the above questions are fluent and without errors. |
|  | Where do you live? | 1 | The responses to the above questions are somewhat fluent; the response time is increased, or 1-2 questions are answered incorrectly. |
|  | What do you do for work? | 2 | There is difficulty in expression, incorrect phrases, or the inability to answer the questions. |
| **2.** **Listening Comprehension Ability: Make judgments based on the following questions.** | Is this month May? | 0 | Completely understood the above questions, with no errors in judgment. |
|  | Do you live in Shanghai? | 1 | Not fully understood the above questions, with 1-2 questions answered incorrectly. |
|  | Are you a doctor? | 2 | Unable to understand the above questions, with all answers incorrect or unable to respond. |
| **3.** **Execution of Instructions: Have the patient execute the following instructions.** | Raise your right hand. | 0 | All of the above instructions can be completed without errors. |
|  | Close your eyes. | 1 | There are understanding barriers; the evaluator needs to provide additional explanations, or 1-2 instructions are executed incorrectly. |
|  | Point at the window, then point at the door. | 2 | Unable to understand the above instructions and cannot execute them. |
| **4.** **Repetition: Have the patient repeat the following content.** | Please turn around and close the door. | 0 | All of the above names can be repeated correctly without errors. |
|  |  | 1 | There are partial barriers in repetition, and prompts are needed during the process. |
|  |  | 2 | There is a complete barrier in repetition, and it cannot be completed. |
| **5.** **Naming: Have the patient name the following items.** | 1.Fountain pen  2.Mobile phone  3.Watch | 0 | All of the above items can be named correctly without errors. |
|  |  | 1 | There are partial barriers in naming ability; the individual can name 1-2 items. |
|  |  | 2 | There is a complete barrier in naming; none of the 3 items can be named. |

**b130 Energy and drive functions**

Item description: General mental functions of physiological and psychological mechanisms that cause the individual to move towards satisfy specific needs and general goals in a persistent manner.

Inclusions: functions of energy level, motivation, appetites, craving (including craving for substances that can be abused), and impulse control

Exclusions: consciousness functions; sleep functions; psychomotor functions; emotional functions

Assessment Prompt: In the last two days, have you felt energetic?

Assessment Guidelines: The assessment can refer to the following content: “Feeling energetic” means having an intrinsic drive and good physical strength to complete daily activities and tasks, being full of energy without feelings of inadequacy or fatigue. The emphasis here is on the patient’s overall feeling of being energetic over the past two weeks. It mainly involves intrinsic motivation, energy levels (appearance), appetite, and the ability to control impulses.

Evaluators can communicate with the patient through the following questions to understand the patient’s energy and drive levels based on their responses.

The score for this item is the total sum of the scores from the following five questions.

| **Question** | **Score** | **Score Prompt** |
| --- | --- | --- |
| 1. Do you want to improve your health level by actively participating in rehabilitation training? | 0 | Very good |
|  | 1 | Average |
|  | 2 | Very poor |
| 2. Do you have enough energy to participate in rehabilitation training? | 0 | Very good |
|  | 1 | Average |
|  | 2 | Very poor |
| 3. Outside of rehabilitation exercises, do you have enough energy to engage in activities you are interested in? | 0 | Very good |
|  | 1 | Average |
|  | 2 | Very poor |
| 4. How is your appetite recently? | 0 | Very good |
|  | 1 | Average |
|  | 2 | Very poor |
| 5. How is your ability to control impulses? | 0 | Very good |
|  | 1 | Average |
|  | 2 | Very poor |

**b280 Sensation of pain**

Item description: An unpleasant subjective feeling related to emotion, specifically referring to persistent pain in a quiet state.

Inclusions: sensations of generalized or localized pain, in one or more body part, pain in a dermatome, stabbing pain, burning pain, dull pain, aching pain; impairments such as myalgia, analgesia and hyperalgesia

Assessment Prompt: In the last two days, have you experienced any pain, and what is the level of that pain?

Assessment Guidelines: The person being assessed should consider the overall level of their pain and its impact on daily life and mark their perception of pain on the following rating scale from 0 to 10 (NRS).

| No pain | 0 1 2 3 4 5 6 7 8 9 10 | Unbearable pain |
| --- | --- | --- |
|  |  |  |

In the pain assessment, consider:

**0 Points:** No pain at all.

**1-3 Points:** Pain is present in daily life but does not affect movement or sleep.

**4-6 Points:** Pain affects movement and sleep, but daily activities can still be completed.

**7-9 Points:** Unable to complete daily activities, showing signs of distress.

**10 Points:** Unbearable pain.

**b134 Sleep functions**

Item description: Can selectively sleep and maintain appropriate time and quality to meet daily needs.

Inclusions: functions of amount of sleeping, and onset, maintenance and quality of sleep; functions involving the sleep cycle, such as in insomnia, hypersomnia and narcolepsy

Exclusions: consciousness functions; energy and drive functions; attention function; psychomotor functions

Assessment Prompt: In the last two days, have you had any sleep problems?

Assessment Guidelines: This assessment includes the quantity of sleep, the onset of sleep, the maintenance of sleep, and the quality of sleep. It addresses sleep disorders such as insomnia, hypersomnolence, and narcolepsy. After considering sleep duration, sleep quality, and the impact of sleep problems on daily life, the person being assessed should mark the corresponding number on the following scale from 0 to 10 (NRS).

| completely normal | 0 1 2 3 4 5 6 7 8 9 10 | complete impairment |
| --- | --- | --- |
|  |  |  |

**b152 Emotional functions**

Item description: The ability of an individual to generate appropriate emotions and manage various different emotions.

Inclusions: functions of appropriateness of emotion, regulation and range of emotion; affect, sadness, happiness, love, fear, anger, hate, tension, anxiety, joy, sorrow; lability of emotion; flattening of affect

Exclusions: temperament and personality functions; energy and drive functions

Assessment Prompt: In the last two days, has your mood affected your daily life or work?

Assessment Guidelines: The assessment involves whether the individual can express appropriate emotions and if there are any emotional distortions; whether they can control and regulate their emotions during feelings of happiness, joy, anger, and sadness; the stability of their mood; and whether there are instances of outbursts, inappropriate language, disordered expressions, physical aggression, or excessive silence. The person being assessed should evaluate their ability to generate, control, and regulate emotions and mark the corresponding number on the following scale from 0 to 10 (NRS).

| completely normal | 0 1 2 3 4 5 6 7 8 9 10 | complete impairment |
| --- | --- | --- |
|  |  |  |

#### Source of Information

The scores for each ICF category are based on the true performance of the individual being assessed, specifically what they are actually able to do at the time of the assessment and in their real environment (which may include positive environmental factors as well as negative ones). For example, in the case of “d465 Moving around using equipment”, if the individual requires a wheelchair or crutches to move, these can be considered positive environmental factors. Therefore, when assessing d465, one should evaluate the individual’s actual performance with the assistance of a wheelchair or crutches. Similarly, for “d550 Eating”, if a patient has the ability to eat and swallow independently, but often requires complete assistance from others during the process, then the score for this item should reflect this need with a value between 7 and 9.

When conducting assessments, the evaluator should utilize all available information, as the focus of attention may vary for different categories, or aspects of function. Some functions can be directly observed, while others cannot and require inference and deduction. In the ICF categories, such as emotional function and pain, data from self-reports can serve as a valid source of information. In some cases, it is necessary to integrate information from multiple sources to discern which information is valid and reliable and which is not. Ultimately, we will rely on the information that has been determined to be true and reliable to assess the score for a particular ICF category. Overall, when assessing functions, we typically have the following sources of information.

7.1 Medical History of the Individual Being Assessed

The medical history information of the individual being assessed can be obtained from their previous medical records or other relevant documentation, as well as through conversations with the individual. For individuals with communication barriers, the evaluator can also gather information by conversing with family members or caregivers of the individual. Medical history information can reflect the issues faced by the individual and their functional level. After effective screening and processing, this information can be used for the assessment of specific items.

7.2 Laboratory or Instrumental Tests and Observations

When evaluating the items, the evaluator can define the extent of disability by conducting objective examinations or observing the individual performing tasks. For instance, when assessing d450 (walking function), the evaluator can understand the degree of walking impairment through the 6-Minute Walk Test. The evaluator can also assess items based on the individual’s performance in executing tasks. For example, when assessing d510 (washing oneself), d530 (using the toilet), d540 (putting on and taking off clothes), and d550 (eating), the evaluator can observe how the individual performs these tasks, determining the degree of functional impairment based on the level of assistance needed.

7.3 Direct Communication with the Individual or Caregiver

Some items in the ICF relate to the individual’s personal feelings and experiences, such as b152 (emotional function) and b280 (pain). When evaluating these items, the evaluator can communicate directly with the individual to inquire about their subjective feelings and experiences related to these items, and assess them based on the individual’s feedback. If the patient is unable to communicate normally due to consciousness disturbances, cognitive impairments, speech disorders, etc., information can be sought from family members or caregivers who are familiar with the patient’s condition. They can provide NRS assessments, which the evaluator will then use to judge the appropriateness of these assessments in relation to the patient’s situation.
